# Miscellaneous Notes

**Published:** 1880-05

**Authors:** 


### MISCELLANEOUS NOTES.

Volcanic eruptions are reported near Brown's Park, Wyoming. —*Cincinnati Commercial.*

It is reported, that about 140 miles north of Cheyenne, rich deposits of silver have been discovered.——According to dispatches in the Cincinnati daily papers, Southern planters are in correspondence with the Chinsee companies at San Francisco, with a view of employing Chinese labor in the cotton fields. This undoubtedly portends great changes in the policy of this country toward Chinese emigration.——A very interesting event, the birth of the first elephant known to have taken place in the history of these animals while in a state of captivity, occured on March 10th, at Philadelphia. The mother is a large black asiatic, 23 years of age; the sire somewhat larger and two years older. The baby is 35 inches in height and weighs 214 lbs. avd. The period of gestation is placed at between 20 and 21 months. A scientific "investigation" by Prof. Leidy and others will develop many points of interest that have long been the subject of conjecture.——Prof. Isaac E. Taylor of Bellevue Hospital, in a paper read before the New York Academy of Medicine, advocates: 1. Flagellation of the child's back previous to its complete delivery, as a preventive of uterine hemorrhage; 2. Flagellation of the abdomen of the women after the delivery of the placenta, as a substitute for the introduction of the hand into the uterine cavity of the uterus.——A young Japanese, Mr. Matsdaira, has invented an instrument for solving problems in trigonometry, thus doing away with the laborious and tedious ramifications of sines and cosines, which have heretofore hampered the calculations of engineers in the progress of their work.——Chinese emigration has taken a strong move towards the North-eastern States and it is predicted, that the Californian view of the Chinese question will make many advocates amongst the hitherto theoretical friends of the Celestial, when the Eastern cities have had practical experience in managing China's teeming thousands. ——A Yankee has invented a method to stamp upon eggs the date of their birth, the moment Mrs. Hen leaves the nest.——Dating from the first of March last, it will be no longer necessary to deposit models in the Patent Office as a condition of procuring patents. The Mt. St. Gothard tunnel is completed. Its length is 9¼ miles, something more than a mile longer than Mt. Cenis and cost but $9,700,000.——The oleo margarine manufacturers are vigorously protesting against the law obliging them to stamp their product with its name.——Edison again comes to the front, this time with a process for separating from "tailings" the remaining gold.——In Cabarus Co., N. C, in July last, a Mr. Harris found a meteorite of over two pounds weight; its value to science is that it is of that rare class which "does not show the Widmanstætten figures or lines indicating the characteristic crystalline structure of meteoric irons."——Statistics in January 1880 show that the Bessemer steel interest of this country now employs $30,000,000 of invested capital; 20,000 workmen; has eleven works; pays $7,500,000 for wages and $8,000,000 for transportation per annum and creates a market for over 800,000 tons of iron, 2,200,000 tons of coal and coke; 600,000 tons of limestone and 1,250,000 tons of iron ore. ——A new Art—Baldheaded persons are recommended by one who knows how it is himself, to have a spider painted on the top of their heads in fly-times.—*Exchange.*——In round numbers, the United States annually sends to England $10,500,000 for tin plate. Why not import the block tin from Cornwall and put the labor on it here?——The failure of the French wine crop has directed foreign attention to the American products in this line.——Naphtha is frequently used for disolving resins in making cheap varnishes, but its odor prevents its use on good work.——The Western rivers are nearly all overflowing their banks; the Mississippi breaking through the levee just above New Orleans. The rainfall since February 1st last at a good many points being fully one half of the annual average.——A New England youth has discovered the true way of storing electricity. He takes the electric *currents* and makes them into jelly, it can then be used as needed.—*Illustrated Scientific News.*

